# The Growing Epidemic of Diabetes Among the Indigenous Population of Canada: A Systematic Review

**DOI:** 10.7759/cureus.36173

**Published:** 2023-03-15

**Authors:** Kaaviya Cheran, Chinmayee Murthy, Elisa A Bornemann, Hari Krishna Kamma, Mohammad Alabbas, Mohammad Elashahab, Naushad Abid, Sara Manaye, Sathish Venugopal

**Affiliations:** 1 General Medicine, California Institute of Behavioral Neurosciences & Psychology, Fairfield, USA; 2 Internal Medicine, California Institute of Behavioral Neurosciences & Psychology, Fairfield, USA; 3 Medicine and Surgery, Universidad Latina de Panama, Panama City, PAN; 4 Internal Medicine and Neurology, California Institute of Behavioral Neurosciences & Psychology, Fairfield, USA; 5 Psychiatry, California Institute of Behavioral Neurosciences & Psychology, Fairfield, USA; 6 Cardiology and Internal Medicine, University of Debrecen, Debrecen, HUN; 7 Radiology, California Institute of Behavioral Neurosciences & Psychology, Fairfield, USA; 8 Rheumatology, King Faisal University, Al-Ahsa, SAU; 9 Neurology, California Institute of Behavioral Neurosciences & Psychology, Fairfield, USA

**Keywords:** prevalence, aboriginal people, indigenous population of canada, diabetes mellitus, diabetes

## Abstract

Diabetes is one of the most well-known and well-researched non-communicable diseases known to humankind. The goal of this article is to show that the prevalence of diabetes is constantly increasing among indigenous people, a major population subgroup in Canada. The Preferred Reporting Items for Systematic Reviews and Meta-Analyses (PRISMA) guidelines were used to conduct this systematic review, and the databases used were PubMed and Google Scholar. Studies that were published in the last 15 years (2007-2022) were selected for this review, and after applying the inclusion and exclusion criteria, screening, and removing duplicates, 10 articles were selected for the final review - three qualitative studies, three observational studies, and four studies without a specified methodology. We used the JBI (Joanna Briggs Institute) checklist, NOS (Newcastle-Ottawa Scale) checklist, and SANRA (Scale for the Assessment of Narrative Review) checklist for quality assessment. We found that all the articles showed that the prevalence of diabetes is increasing in all the Aboriginal communities despite all the interventional programs already in place. Rigorous health plans, health education, and wellness clinics for primary prevention can all be effective in reducing the potential risks of diabetes. More studies exploring the prevalence, effects, and outcomes of diabetes in the indigenous population of Canada are needed to effectively understand the disease and its complications in this group.

## Introduction and background

Sir Frederick Banting was a Canadian scientist who discovered insulin and its therapeutics in 1921 in Toronto, Canada. He said, “insulin does not belong to me. It belongs to the world” [1] with the hope that his discovery will reach the entire population of the world and help people manage diabetes. Unfortunately, the Canadian indigenous people are among the population of the world that has a high prevalence of diabetes and does not get adequate medical attention.

Indigenous people, also called Aboriginal people, are the original or native people of North America. The Canadian constitution recognizes three main groups of indigenous people - Inuit, Metis, and Indians, commonly called First Nations. Although there are many countries in the world with significant indigenous populations, the Canadian indigenous population remains to be one of the largest in the world [2]. As of 2021, there are more than 1.8 million people identifying themselves as Canadian Aboriginal [3]. It is also the fastest-growing population in Canada, with an increase of almost 10% in the last five years, whereas the non-indigenous population grew by only 5.3% [2]. Nearly 66% of the indigenous population are in the working age group with an average age of 33.6 years as compared to the non-indigenous population with an average age of 41.8 years [2].

Even though the indigenous population is the youngest population in Canada, it bears the burden of several chronic diseases, with the most devastating being diabetes mellitus [4]. There is a shift in disease patterns from infectious diseases being more prevalent in the past to the recent increase in chronic diseases. This is the result of physical, social, and genetic factors among the Aboriginal communities [4,5].

Diabetes mellitus is one of the most common and prevalent chronic diseases in the world. According to the World Health Organization (WHO), around 422 million people in the world are affected by diabetes [6]. About 1.5 million people die each year due to the complications of diabetes [6]. In Canada, 5.7 million people have been diagnosed with diabetes, although people living with undiagnosed diabetes and prediabetes, a condition that can progress to diabetes, are estimated to be much higher. Among the indigenous population, 17.2% of First Nations living on-reserve, 12.7% of First Nations living off-reserve, 4.7% of Inuit people, and almost 10% of Metis people have been affected by diabetes [7].

Diabetes has many subtypes with the most common being type 2 diabetes mellitus. It is a metabolic disorder that arises due to either deficiency or insensitivity to insulin, a hormone that is involved in blood glucose regulation. As a result of this, there is an increase in the blood glucose concentration that can lead to multi-organ failure and damage to the peripheral nerves, if left untreated. The purpose of this review is to examine the current trend of all forms of diabetes, summarize the available literature, and show that the prevalence of diabetes is increasing among the Aboriginal population of Canada.

## Review

Methods

The Preferred Reporting Items for Systematic Reviews and Meta-Analysis (PRISMA) guidelines were used to do a systematic literature search [8].

Search Strategy and Databases

The databases used for this systematic review are PubMed, Web of Science, ScienceDirect, and Google Scholar. The following Medical Subject Headings (MeSH) were used in PubMed: "Indigenous Canadians"[MeSH] and "Diabetes Mellitus"[MeSH] alone and in combination. Regular keywords such as “Diabetes mellitus,” “Diabetes,” “Type 2 diabetes mellitus,” “Indigenous people,” “Canadian Aboriginals,” “Canadian Indigenous,” “First Nations,” “Metis,” and “Inuit” were also used. The search strategy is illustrated in Table 1. 

**Table 1:** Search strategies MeSH, Medical Subject Headings

S. No.	Databases	MeSH combination and keywords	Search results
1.	PubMed	"Indigenous Canadians"[MeSH] AND "Diabetes Mellitus"[MeSH]	180
2.	Google Scholar	“Diabetes Mellitus” OR “Diabetes” OR “Type 2 Diabetes” AND “Indigenous Canadians” OR “Canadian Aboriginals” OR “First Nations” OR “Metis” OR “Inuit”	29
3.	Web of Science	"Diabetes" OR "Diabetes Mellitus" OR "Type 2 Diabetes" (Topic) AND "Indigenous Canadians" OR "Aboriginal Canadians" (Topic)	52
4.	ScienceDirect	“Diabetes Mellitus” OR “Diabetes” OR “Type 2 Diabetes” AND “Indigenous Canadians” OR “Canadian Aboriginals” OR “First Nations” OR “Metis” OR “Inuit”	193

Eligibility Criteria

All studies that highlight diabetes mellitus in the Canadian indigenous population and were published between 2007 and 2022 were included. Articles that discussed all age groups, both genders, human species, and articles in the English language were all a part of our inclusion criteria. Non-English literature and abstract-only papers were excluded.

Screening and Quality Assessment

The first and second authors of this article screened titles and abstracts of all the articles. Screening of the full text of the shortlisted articles was then performed, and the final list of articles for this review was chosen. We then compiled the data retrieved from all the papers, and its quality was assessed individually.

The Joanna Briggs Institute (JBI) checklist was used to assess the quality of quantitative studies; a score above 7 was considered high quality, and a score below 4 was considered low quality.

The Newcastle-Ottawa Scale (NOS) checklist was used to assess the quality of observational studies; a score above 7 was considered a low risk of bias and hence high quality, and a score of less than 3 was considered a high risk of bias and low quality.

The Scale for the Assessment of Narrative Review Articles (SANRA) checklist was used to assess the quality of narrative review papers and those that did not have a clear methods section. Articles that satisfied more than 60% of the checklist were considered high quality. The quality assessment tools used for the various study types are shown in Table 2. ​​​

**Table 2:** Quality assessment tools used for this systematic review JBI, Joanna Briggs Institute; NOS, Newcastle-Ottawa Scale; SANRA, Scale for the Assessment of Narrative Review

Study type	Number of studies	Quality assessment tool
Qualitative study	5	JBI checklist
Observational study	3	NOS checklist
Studies without clear methods section	5	SANRA checklist

Results

**Figure 1:**
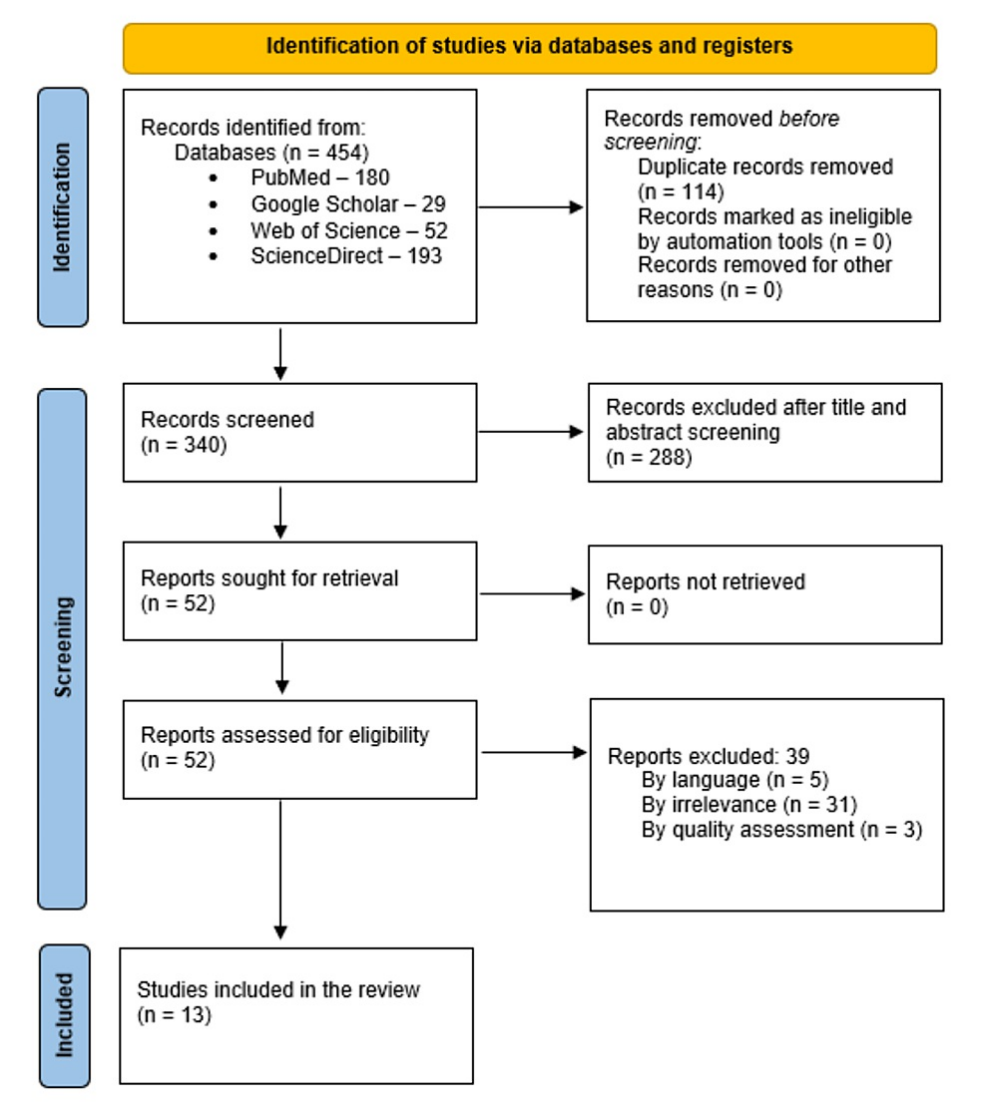
PRISMA flowchart PRISMA, Preferred Reporting Items for Systematic Reviews and Meta-Analyses

Our literature search of the databases yielded a total of 454 articles - 180 articles in PubMed, 52 articles in Web of Science, 193 articles in ScienceDirect, and 29 articles in Google Scholar. After removing the duplicates, we were left with 340 articles, and the inclusion and exclusion criteria were used to review the articles. Then, the first and second authors proceeded to screen the titles and abstracts of the articles. The screening led to the exclusion of 288 articles, and the remaining 52 articles were sought for retrieval. As we were able to retrieve all 52 articles, these were assessed for eligibility for this review. Out of these, 39 articles were excluded as they did not meet the criteria of the study. Finally, a total of 13 articles were selected for this systematic review as shown in the following PRISMA flowchart, Figure 1. 

The results of the quality assessment were as follows: out of the five qualitative studies used in this systematic review, three articles scored 8 out of 10 on the JBI checklist and two articles scored 7 out of 10. The NOS used for the three observational studies showed that one study scored 7 out of a maximum of 9 and two studies scored 9 out of 9. The five studies without a clear methods section scored 10 out of a possible 12 on the SANRA checklist.

The type and purpose of each study that is included in the systematic review are shown in the following table, Table 3, along with the conclusion of the study.

**Table 3:** Study characteristics and the data extracted from the selected studies

S. No.	Author and year of study	Type of study	Purpose of study	Conclusion
1.	Bruce et al., 2014 [4]	Survey Research	To compare chronic conditions like diabetes in the Aboriginal population	The Aboriginal population of Canada has heterogenous health status, and special surveillance is required to treat this population
2.	Matsumoto et al., 2020 [9]	Retrospective Study	To examine the prevalence and epidemiology of diabetes in 25 First Nations communities in Northwest Ontario	The diabetes prevalence in these communities is twice of the non-indigenous population
3.	Wicklow et al., 2021 [10]	Qualitative Study	To determine the experience of indigenous adolescents living with diabetes as the incidence continues to rise	A broader understanding of their experience is needed to improve the provision of healthcare
4.	Oster et al., 2011 [11]	Longitudinal Study	To compare the incidence and prevalence of diabetes among Aboriginal adults and adults in the general population in Alberta	The incidence and prevalence of diabetes were higher in the Aboriginal population than in the general population
5.	Hummelen et al., 2020 [12]	Retrospective Study	To examine the prevalence and birth outcomes of diabetes in pregnancy in First Nations hospitals	Patients with diabetes in these hospitals vary substantially from non-indigenous patients and so resources must cater to their specific needs
6.	Vélez et al., 2020 [13]	Cohort Study	To examine temporal trends and utilization of healthcare services by First Nations women with diabetes during pregnancy	Disparities in outcomes between First Nations women and other women highlight the need for specialized care
7.	Batal et al., 2021 [14]	Survey Research	To describe the health status, diabetes prevalence, and obesity among First Nations living on reserve	Higher prevalence of diabetes and obesity were identified in First Nations, and a better understanding of their concerns is needed to identify solutions
8.	Murdoch-Flowers et al., 2019 [15]	Survey Research	To examine if culturally based interventions reduce the incidence of diabetes in Mohawk territory, Canada	Culturally appropriate health education can help to create healthy changes in people with chronic conditions like diabetes mellitus
9.	Pelletier et al., 2012 [16]	Report Summary	Diabetes surveillance report published by the Public Health Agency of Canada	Compared to the non-Aboriginal population, diabetes was diagnosed at a younger age in the Aboriginal population who experienced higher complications
10.	Bird et al., 2008 [17]	Qualitative Study	To know the experiences of Inuit people living with diabetes	The current health prevention services may be insufficient to treat the Inuit people with diabetes
11.	Elamurugan et al., 2022 [18]	Qualitative Study	To explore the social determinants that lead to increased incidence of gestational diabetes in the indigenous population	More community-driven efforts are needed to address the issue effects of gestational diabetes in this population
12.	Voaklander et al., 2020 [19]	Qualitative Study	To compare the prevalence of preexisting diabetes and gestational diabetes between indigenous women and non-indigenous women	Both conditions were more common in indigenous women when compared to non-indigenous women
13.	Morriseau, 2022 [20]	Editorial Article	To give a perspective on diabetes in the indigenous population and inequalities in health outcomes	The disparities in diabetes between indigenous and non-indigenous population will increase if changes to research and clinical care are not made

Out of the 13 studies selected in this systematic review, three studies showed that a broader and better understanding of the health needs of the Aboriginal people is needed to be able to help these communities better. Four studies showed that specialized care that can be customizable for individual communities is required as their health needs differ from that of the general population. Two studies demonstrated that the current healthcare plan in place is not enough to support the health of the Inuit people. Six of these studies summarized that the prevalence of diabetes is more in the indigenous population when compared to the general population, and one of these studies even showed that the incidence of diabetes was at a younger age and the complication rates were higher in these communities. 

Discussion

To examine the prevalence of diabetes in the Aboriginal population of Canada, we selected 13 previously published articles and studied them. These articles that were published from 2007 to 2022 show that the incidence and prevalence of diabetes in the Aboriginal population have been steadily increasing from 2007 till date despite the public health measures in place.

Disparities in Age and Gender

In a recent study by Matsumoto et al., published in 2020, it was shown that the overall age-adjusted prevalence of diabetes among the 25 First Nations communities in Northwest Ontario was 15.1%, while the prevalence in the general population in 2017 was 8.8% [9]. The age of onset of diabetes in the indigenous population is much lower than that of the general population. Aboriginal adolescents are disproportionately affected by diabetes, and because of this, the prevalence of diabetes in this population is much higher [9-11]. It was also noted that within the same community, women had a higher prevalence of diabetes when compared to men, after the age of 30. This is in contrast with what is seen in the general population of Canada, in which there is a male predominance of diabetes [9,11,12]. This disproportionate increase in the prevalence of diabetes in women of reproductive age could be due to the prevalence of obesity among women of reproductive age. Another important reason is that the higher prevalence of gestational diabetes (GDM) among this population brings in a whole group of complications in these women [18,19]. The maternal and neonatal adverse effects in diabetes complicating pregnancy are numerous, some of which are unplanned C-sections, stillbirths, congenital anomalies, macrosomia, and neonatal hypoglycemia. It was observed that these adverse perinatal outcomes were more commonly seen in a hospital serving the Aboriginal people in Northwest Ontario [12]. A study by Vélez et al., published in 2020, provides data showing that the Aboriginal communities also have the burden of undiagnosed type 2 diabetes in women of reproductive age that becomes apparent only during their first prenatal visit to the obstetrician [13].

What Causes the Increased Prevalence of Diabetes?

The risk factors for the increased prevalence of diabetes in the indigenous population of Canada are listed in Table 4 along with the points to consider for each risk factor. 

**Table 4:** Summary of probable factors leading to increased prevalence of diabetes

S. No	Risk factors	Points to consider
1.	Genetic and biological	Higher obesity rates, higher gestational diabetes rates leading to increased risk of diabetes and increased risk of obesity in offspring, impaired glucose tolerance in females, and thrifty gene theory
2.	Environmental	Less access to healthcare services, remote geographical location, reduced availability of healthy food supply, less education opportunities, and hunting and fishing restrictions leading to less traditional food
3.	Lifestyle	Smoking, energy-dense westernized food leading to obesity and increased body mass index (BMI), and less physical activity - hunting restrictions
4.	Sociocultural	Lack of culturally appropriate support, language barriers, mistrust of outsiders, political and historical factors such as the residential school system, and loss of native land
5.	Economic	Lack of employment, cost of daily diabetes care, expensive healthy food options, and transportation costs to access healthcare

The factors that contribute to the increased prevalence of diabetes in the indigenous population are multiple in number and complex in nature. Canadian Aboriginal people have been subjected to centuries of colonization and marginalization. The concept of "ecological grief" has been established among the indigenous population [14]. This concept explains that the loss of land and destruction of indigenous habitats has led to a decline in the general health of this population. The Aboriginal people have a holistic concept of health comprising emotional and spiritual health along with physical and mental health [21]. Any factor that can disrupt these dimensions can be detrimental to the health of these people. The loss of traditional lifestyles and food habits and the westernization of diets have led to an increase in the prevalence of obesity, which is a proven risk factor for the development of type 2 diabetes [14,15,18]. The prevalence of diabetes is the lowest in the Inuit (4.7%) among all the indigenous communities, and this can be due to the fact that these people religiously follow the traditional living methods to date [4,5,22]. Lack of education, poverty, and substandard living conditions due to lack of income are all well-established causes of poor health. Since the indigenous people are all affected by at least one of the above factors, chronic diseases like diabetes are seen more commonly in these communities. The Aboriginal people who live within their community in the Aboriginal land are known as people living on-reserve, and those people who identify as indigenous but move out of their land to other towns or cities are people living off-reserve. The Aboriginal people, mainly the younger population, move out of their communities for better education, job opportunities, and a better life. It was found that there were significant disparities in health between the indigenous people that live on the reserve when compared to people that live off the reserve. The prevalence of diabetes among people living on-reserve is estimated to be 17.2% and those that live off the reserve is estimated to be 10.3% [23]. In a study by Park et al., in 2015, it was shown that income and education were the causes of disparities in the avoidable mortality measure due to diabetes, among the indigenous and non-indigenous populations of Canada [24]. People who held regular jobs and received wages were less commonly diagnosed with diabetes [14], and another study showed that indigenous people with diabetes considered traditional or healthy food options expensive and required financial assistance to eat healthy [18,22].

Sociocultural Factors

Many studies that were included in this review show a strong correlation between the sociocultural factors of a community and diabetes. The Wicklow et al. study in 2021 reported that patients described diabetes as a "sociocultural disease" that was influenced by their emotional and cultural environment [10]. Although there may be similarities in disease profile and impact on the various indigenous communities at first glance, in-depth research shows that the three main communities - First Nations, Inuit, and Metis - all have different histories and cultural differences, and these affect the disease outcomes in each of these communities and should be taken into considerations when planning interventions [4]. Another concept specific to the indigenous communities called "cultural continuity" has been described in the study by Oster et al. The communities that did not lose their identity and followed their cultural and traditional lifestyles strictly were relatively protected from diabetes and other chronic diseases when compared to the communities that moved toward westernization [25].

Complications of Diabetes

Diabetes mellitus is a chronic condition that causes an increased rate of morbidity and mortality in those that are afflicted by it. The study by Matsumoto et al. shows that indigenous women who had GDM had a greater transition to type 2 diabetes mellitus later in life when compared to the general population [9]. The Aboriginal adolescent population expressed that living with diabetes was a "burden," was confusing, and complicated their everyday life [10]. They were living with the constant fear of the complications of diabetes like infections, diabetes nephropathy, and loss of limbs. The Matsumoto et al. study also shows that these communities have higher rates of lower limb amputation, almost seven-fold more and twice the rate of advanced kidney disease, due to diabetes than the general population [9].

Challenges in Management of Diabetes

Management of diabetes is complex and management in indigenous communities has its own barriers. Colonization of the Aboriginal land and years of exploitation have led to the mistrust of outsiders in the Aboriginal communities. These communities, especially those that live on the reserve, view new health programs with skepticism [17]. They do not readily accept health educational sessions or primary prevention programs. There are many programs for financial aid for indigenous communities, but even with these, there are other barriers to accessing healthcare such as their geographic location and transportation facilities, especially during Canadian harsh winters [26]. Even though the number of visits to a primary care physician was adequate, access to specialized care was limited [13]. Diabetes is a complex disease affecting many parts of the body, and specialists such as endocrinologists, ophthalmologists, and podiatrists are needed for the adequate management of diabetes. Self-management of diabetes also has some complications. Gaps in the knowledge about blood sugar levels, medications, healthy food choices, and daily foot surveys make it difficult for these people to manage diabetes [17]. Prescription medication costs are covered through insurance for the First Nations with treaty status, but other costs associated with the self-care of diabetes such as blood sugar testing strips and lancets were not covered, deterring these patients from taking care of themselves [26].

Education and Support

Diabetes is a multifaceted disease, and its management needs to consider all the factors. Pharmaceutical intervention is only a part of the treatment, and a major aspect of management is to provide social support to the people affected by diabetes. This could be in the form of financial support for self-management, providing information and education about diabetes, and emotional or mental support. In one study with adolescents, a study by Wicklow et al., 2021, the patients explained the importance of social support to effectively manage diabetes [10]. Culturally appropriate education will equip the Aboriginal people with knowledge about their disease and the different management options. The difference in the prevalence of diabetes between the Aboriginal population and the general population of Alberta appears to be less pronounced than the difference found in other provinces. It has been speculated to be due to community-based educational programs in addition to federal programs [11].

Cost of Diabetes

Another aspect of diabetes that needs to be addressed is the economic impact it has on healthcare and its costs. In general, the healthcare costs for the management of diabetes are estimated to be $30 billion a year [7]. A Manitoba study estimated that the increased prevalence of diabetes in the indigenous population added to the healthcare expenses by 15.9%, almost $7.4 million, and the increased healthcare utilization costs were estimated to be 14.6%, nearly $6.8 million [27]. This was attributed to the First Nations people being 2.5 times more likely to be admitted to the hospital for complications of diabetes such as amputations, cardiac complications, or dialysis when compared to the general population with diabetes. Another study in Saskatchewan showed that the First Nations people with diabetes were 1.8 times more likely to be hospitalized due to complications than the general population with diabetes. This article also shows that the healthcare costs for the indigenous population with diabetes were 2.3 times higher than the costs for the general population with diabetes, and this increase in costs was due to hospitalization, specialist visits, and dialysis [27].

Limitations

This systematic review has certain limitations. The indigenous population of Canada is a less studied population, and many of the studies focused on the First Nations. We couldn’t find many studies that focused on diabetes in the Metis and Inuit indigenous groups. Languages other than English were one of our exclusion criteria and so we may have lost important relevant articles, especially those published in French. More research into the lives of the indigenous population exploring the various determinants of their health keeping in mind the various challenges is recommended for the future to put an end to the health inequalities faced by this population.

## Conclusions

This review focuses on diabetes and its effects on the indigenous population of Canada. It shows that the indigenous population is disproportionately affected by diabetes. It highlights the need for culturally appropriate education and intervention for diabetes. Recent trends show that interventions that closely relate to the traditional lifestyle of the indigenous population and community-based support may have a positive shift in the health of these people. Involving the Aboriginal people and listening to their opinion can help to construct successful health promotion programs within the community. This review also highlights the financial burden of diabetes in the Aboriginal population, the increasing healthcare costs for the government, and the need for additional research to tackle these issues. Even though diabetes is an extensively researched disease, diabetes among the indigenous population remains a less studied area. These people have always displayed tremendous strength and resilience in facing adversities including chronic diseases, and exploring the history, genetics, and environment of the Aboriginal people can equip the healthcare system with the knowledge to fight against diabetes in this population.

## References

[1] Das T, Rani PK, Sivaprasad S, Raman R (2021). The blue circle and 100 years of insulin discovery. Indian J Ophthalmol.

[2] (2022). Statistics Canada. http://www150.statcan.gc.ca/n1/daily-quotidien/220921/dq220921a-eng.htm.

[3] (2022). Statistics Canada. 2022. Census Profile, 2021 Census of Population.. https://www12.statcan.gc.ca/census-recensement/2021/dp-pd/prof/index.cfm?Lang=E.

[4] Bruce SG, Riediger ND, Lix LM (2014). Chronic disease and chronic disease risk factors among First Nations, Inuit and Métis populations of northern Canada. Chronic Dis Inj Can.

[5] (2022). Diabetes Canada. https://www.diabetes.ca/resources/tools---resources/indigenous-communities-and-diabetes.

[6] (2022). World Health Organization. https://www.who.int/health-topics/diabetes.

[7] (2022). Diabetes Canada, press releases. https://www.diabetes.ca/media-room/press-releases/diabetes-rates-continue-to-climb-in-canada.

[8] Page MJ, McKenzie JE, Bossuyt PM (2021). The PRISMA 2020 statement: an updated guideline for reporting systematic reviews. BMJ.

[9] Matsumoto CL, Tobe S, Schreiber YS (2020). Diabetes prevalence and demographics in 25 First Nations communities in Northwest Ontario (2014-2017). Can J Rural Med.

[10] Wicklow B, Dart A, McKee J, Griffiths A, Malik S, Quoquat S, Bruce S (2021). Experiences of First Nations adolescents living with type 2 diabetes: a focus group study. CMAJ.

[11] Oster RT, Johnson JA, Hemmelgarn BR (2011). Recent epidemiologic trends of diabetes mellitus among status Aboriginal adults. CMAJ.

[12] Hummelen R, Kattini R, Poirier J, Madden S, Ockenden H, Dooley J, Kelly L (2020). Demographics, prevalence and outcomes of diabetes in pregnancy in NW Ontario. Can J Rural Med.

[13] Vélez MP, Slater M, Griffiths R (2020). Diabetes during pregnancy and perinatal outcomes among First Nations women in Ontario, 2002/03-2014/15: a population-based cohort study. CMAJ Open.

[14] Batal M, Chan HM, Fediuk K, Ing A, Berti P, Sadik T, Johnson-Down L (2021). Associations of health status and diabetes among First Nations Peoples living on-reserve in Canada. Can J Public Health.

[15] Murdoch-Flowers J, Tremblay MC, Hovey R, Delormier T, Gray-Donald K, Delaronde E, Macaulay AC (2019). Understanding how indigenous culturally-based interventions can improve participants' health in Canada. Health Promot Int.

[16] Pelletier C, Dai S, Roberts KC, Bienek A, Onysko J, Pelletier L (2012). Report summary. Diabetes in Canada: facts and figures from a public health perspective. Chronic Dis Inj Can.

[17] Bird SM, Wiles JL, Okalik L, Kilabuk J, Egeland GM (2008). Living with diabetes on Baffin Island: Inuit storytellers share their experiences. Can J Public Health.

[18] Elamurugan K, Esmaeilisaraji L, Strain J (2022). Social inequities contributing to gestational diabetes in indigenous populations in Canada: a scoping review. Can J Diabetes.

[19] Voaklander B, Rowe S, Sanni O, Campbell S, Eurich D, Ospina M (2020). Prevalence of diabetes in pregnancy among indigenous women in Australia, Canada, New Zealand, and the USA: a systematic review and meta-analysis. Lancet Glob Health.

[20] Morriseau TS (2022). Determinants of wellness: a perspective on diabetes and indigenous health. Can J Diabetes.

[21] Halseth R (2019). The Prevalence of Type 2 Diabetes Among First Nations and Considerations for Prevention. Prince George, BC: National Collaborating Centre for Aboriginal Health.

[22] Rosella LC, Kornas K, Green ME, Shah BR, Walker JD, Frymire E, Jones C (2020). Characterizing risk of type 2 diabetes in First Nations people living in First Nations communities in Ontario: a population-based analysis using cross-sectional survey data. CMAJ Open.

[23] Crowshoe L, Dannenbaum D, Green M, Henderson R, Hayward MN, Toth E (2018). Type 2 diabetes and indigenous peoples. Can J Diabetes.

[24] Park J, Tjepkema M, Goedhuis N, Pennock J (2015). Avoidable mortality among First Nations adults in Canada: a cohort analysis. Health Rep.

[25] Oster RT, Grier A, Lightning R, Mayan MJ, Toth EL (2014). Cultural continuity, traditional indigenous language, and diabetes in Alberta First Nations: a mixed methods study. Int J Equity Health.

[26] Kulhawy-Wibe S, King-Shier KM, Barnabe C, Manns BJ, Hemmelgarn BR, Campbell DJT (2018). Exploring structural barriers to diabetes self-management in Alberta First Nations communities. Diabetol Metab Syndr.

[27] (2022). Diabetes care and management in indigenous populations in Canada - summary report of a pan-Canadian policy roundtable November 1, 2017. https://era.library.ualberta.ca/items/6b795abc-533b-4171-9244-02b0985094ed.

